# Implementation of diabetic retinopathy screening in the Oslo region, Norway: a 3-year pilot study

**DOI:** 10.3389/fmed.2026.1748289

**Published:** 2026-02-24

**Authors:** Ellen Steffenssen Sauesund, Cathrine Brunborg, Øystein Kalsnes Jørstad, Morten Carstens Moe, Maja Gran Erke, Dag Sigurd Fosmark, Goran Petrovski

**Affiliations:** 1 Department of Ophthalmology, Oslo University Hospital, Oslo, Norway; 2 Center for Eye Research and Innovative Diagnostics, Department of Ophthalmology, Institute of Clinical Medicine, Faculty of Medicine, University of Oslo, Oslo, Norway; 3 Oslo Centre for Biostatistics and Epidemiology, Research Support Services, Oslo University Hospital, Oslo, Norway; 4 Norwegian Directorate of Health, Oslo, Norway; 5 School of Medicine, University of Split, Split, Croatia; 6 UKLONetwork, University St. Kliment Ohridski-Bitola, Bitola, North Macedonia

**Keywords:** albuminuria, diabetic retinopathy screening, disease progression, population-based pilot study, risk stratification

## Abstract

**Purpose:**

Determine the prevalence and progression of diabetic retinopathy (DR) and its risk factors over a three year period in a pilot DR screening program in the Oslo region.

**Methods:**

The pilot screening program enrolled 90 adult patients with type 1 (T1D) or type 2 diabetes (T2D) from December 2019 to January 2021. Patients were referred by general practitioners and underwent annual DR screening, including fundus photography and optical coherence tomography (OCT) imaging. Baseline and follow-up data included socio-demographic parameters, diabetes history and medication, glycated haemoglobin (HbA1c), visual acuity, blood pressure, and intraocular pressure. DR severity was graded using the International Clinical Disease Severity Scale for DR, and diabetic macular edema (DME) was identified based on OCT findings. Cox regression analyses were conducted to identify factors associated with DR progression.

**Results:**

At baseline, prevalence of DR was 27.8%, with 6.7% of the patients exhibiting vision-threatening DR (VTDR). Over the 3-year follow-up, 24.7% of the patients either developed or showed a progression of DR, with 4% developing VTDR. Duration of diabetes and urine albumin-to-creatinine ratio were significantly associated with DR progression (hazard ratio 1.07 (95% confidence interval 1.02–1.12) and 1.03 (95% confidence interval 1.01–1.06), respectively). 13.3% of patients were lost to follow-up, primarily due to the COVID-19 pandemic.

**Conclusion:**

Most patients had stable DR over the 3 years period. Diabetes duration and albuminuria predicted progression, supporting extended screening intervals for low-risk patients with risk-based stratification.

## Introduction

Diabetic retinopathy (DR) is a frequent complication of diabetes mellitus (DM) (1, 2). It is one of the leading contributors to global blindness in persons aged 50 years and above and constitutes a main cause of vision impairment in those aged 25–74 years (3, 4). DR often remains asymptomatic until advanced disease has developed, which underscores the importance of systematic DR screening to detect and treat sight-threatening complications. Early detection of DR by regular screening is shown to prevent vision loss (5–7). Globally, there are different approaches to DR screening, with variations in the availability of diverse imaging technology. Numerous studies have verified the cost-effectiveness of DR screening programs (6, 8–11). Norway has not had a systematic national screening program for DR, and most DR patients are followed up by either contracted specialists/ophthalmologists or at the hospital without a centralized register. Consequently, knowledge regarding the prevalence of DR, as well as associated risk factors among people with diabetes in Norway, remains limited. It is estimated that approximately 316,000–345,000 individuals in Norway are living with DM, of whom around 60,000 are undiagnosed and approximately 90% have type 2 diabetes (T2D) (12, 13). Baseline results of this current pilot study have been published in two cross-sectional studies (14, 15), which revealed a prevalence of 58.3 and 23.1% for any DR in patients with type 1 diabetes (T1D) and T2D, respectively (14), which is consistent with another Norwegian study (16). Additionally, lack of ophthalmological examinations and follow-ups for a considerable number of patients was detected (14).

Worldwide, there are many cross-sectional studies on DR screening, but there is a shortage of follow-up studies. In low income countries, the lack of such studies could be related to poor attendance and follow-up due to financial factors, but also educational, socio-cultural and health system-related factors besides poor infrastructure for transportation (17, 18).

Recently, the Norwegian health authorities decided to formally develop a nationwide DR screening program. To better understand the local burden of disease, the Department of Ophthalmology in Oslo, Norway initiated a pilot DR screening program. This program aimed to evaluate the prevalence and progression of DR and to identify associated risk factors among people with DM in the Oslo region. The present study reports the 3-year results of this pilot screening program.

## Materials and methods

### Study design and population

A prospective DR screening study in adults (≥18 years of age) with T1D or T2D was conducted. 90 patients affiliated with Oslo University Hospital (OUH), Norway, where the study took place, were included. The patients were enrolled from December 2019 to January 2021.

### Follow-up strategy and referral criteria

General practitioners (GPs) were informed about the project and referred patients in a consecutive manner, while some patients were referred from another affiliated healthcare institution. The GPs were invited to refer patients without any known treatment-dependent DR and who were not already being followed-up by an ophthalmologist. The patients were followed up for 3 years with annual examinations. In some few cases, more frequent examinations were implied if clinically necessary (e.g., pregnancy or uncertainty regarding the DR being vision-threatening).

### Inclusion and exclusion criteria

Inclusion criteria were adults (≥18 years of age) with T1D or T2D affiliated with OUH without known treatment-dependent DR and not already being followed-up by an ophthalmologist. As a screening study, we did not exclude any of these patients referred, but if the examination revealed VTDR the patient was followed-up in the clinic and not further enrolled in the screening program.

### Outcome definitions

The primary outcome of the study was the development of VTDR, in which case the patients were followed-up in the clinic. Secondary outcomes were the prevalence of DR detected throughout the screening program, the progression from no DR or any DR to a higher grade, or diabetes macular edema, and the identification of risk factors associated with the presence and progression of DR.

### Screening procedures and imaging protocol

At baseline a slit-lamp examination and dilated fundus microscopy was performed. Best-corrected visual acuity (BCVA) was assessed using the Clear Chart 2 (Reichert Technologies, Depew, NY, USA) digital acuity test, which displays five letter optotypes per line and a logarithm of the minimal angle of resolution (logMAR) line size progression (i.e., each letter has a score of 0.02 logMAR). After 1 min of rest, blood pressure (BP) and heart rate (HR) were measured in the left overarm using a calibrated automatic BP monitor (Riester, ri-champion N Automated Blood Pressure Monitor, Jungingen, Germany). Hypertension (HT) was defined as hypertension grade 2 according to the American Heart Association: systolic at least 140 or diastolic at least 90 mm Hg (≥140/90 mmHg). The intraocular pressure (IOP) was measured using an iCare 100 tonometer (Icare Finland Oy, Vantaa, Finland). Mean ocular perfusion pressure (MOPP) was calculated as two-thirds of the systemic mean arterial pressure (MAP) minus the IOP (16). The MAP = diastolic blood pressure (DBP) + one-third (systolic blood pressure (SBP) − DBP). If not available from the referring instance, the following laboratory tests were performed at the department: glycated haemoglobin (HbA1c), total serum cholesterol and urine-albumin, creatinine- and albumin-to-creatinine- ratio (u-ACR). Albuminuria was defined as u-ACR ≥ 3 mg/mmol.

Color fundus photography was conducted using a CLARUS™ 700 camera, Zeiss (Carl Zeiss Meditec AG, Jena, Germany) with fovea- and optic-disc-centered photos, both 133°-field images. OCT macula radial scans of 6 × 6 mm were obtained using NIDEK RS-3000 Advance OCT Retina Scan (NIDEK CO., LTD, Gamagori, Japan).

The fundus images were graded according to the International Clinical Disease Severity Scale for DR (ICDSS) (17): no DR, mild NPDR, moderate NPDR, severe NPDR or proliferative DR. Both eyes were graded through consensus between two experienced ophthalmologists (ES and DF); the eye with the more severe DR defined the individual grade. Diabetic maculopathy based on fundus photography was classified as follows: no diabetic maculopathy (0), presence of microaneurysm(s) within one disc diameter from the foveola (1) and hard exudate(s) within one disc diameter from the foveola (2). DME was in this pilot study defined as the presence of intraretinal cysts and/or subretinal fluid in the central area of the macula, within a distance of 1.5 mm from the foveal center. This was determined qualitatively in our study by two experienced ophthalmologists (ES, DF). VTDR was defined as severe NPDR, proliferative DR, and/or DME, in accordance with ICDSS classification for vision-threatening stages.

Socio-demographic parameters, such as gender, age, type of DM, duration of DM, use of tobacco and alcohol, body weight and body height [used to determine body mass index (BMI)], were registered. Medication history (type of DM medication, duration of insulin treatment and use of cholesterol lowering- and antihypertensive- drugs) was also documented, as well as previous history regarding DR screening.

In the follow-up examinations, fundus photography and OCT were captured and evaluated, accordingly. Again, HbA1c, visual acuity, IOP, HR, BP and body weight were measured and the use of tobacco and alcohol, duration of DM, type of DM medication, duration of insulin treatment, use of cholesterol lowering- and antihypertensive drugs were registered.

### Statistical analyses

No *a priori* sample size calculation was performed. The sample size was determined based on pragmatic considerations like referral capacity and time frame, rather than on formal statistical power calculations.

Descriptive statistics were used to summarize the baseline characteristics of the study population, including demographic data, medical history, and disease status. Continuous variables were presented as means with standard deviations (SD) or medians with interquartile ranges (IQR), depending on the data distribution, while categorical variables were presented as frequencies and percentages. The Mann–Whitney U test was used to investigate differences between two groups for continuous variables and the Chi-square or Fisher’s exact test to detect associations between categorical variables.

The Kaplan–Meier method was used to estimate the time to progression or development of DR, and differences in survival curves were evaluated using log-rank tests. Cox regression analysis was employed to examine the association between clinical variables and the risk of DR progression, estimating hazard ratios (HR) with 95% confidence intervals (CI). The variables analyzed in the Cox regression model included age, sex, HbA1c, duration of diabetes, urine albumin-to-creatinine ratio, hypertension, and other relevant covariates. A 5% significance level was used. We used IBM SPSS Statistics 28.0 (IBM Corp., Armonk, NY, USA) for data analyses.

### Ethics statement

The Regional Committee for Medical and Health Research Ethics concluded that the project was outside the remit of the Norwegian Health Research Act (reference: 28857). The Institutional Data Protection Officer at OUH approved the study (reference: 20/00571). Written informed consent was obtained from all participants.

## Results

The study enrolled 90 patients (180 eyes). Twelve patients (13.3, 95% CI: 7.8, 21.9) had T1D and 78 (86.7, 95% CI: 78.1, 92.2) had T2D. A total of 47 (52.2%) patients were newly diagnosed with DM (e.g., time since diagnosis being <5 years for patients with T1D, and <1 year for patients with T2D). The gender distribution was 61 males (67.8%) and 29 females (32.2%). At baseline, 25 (27.8%) patients had some degree of DR. The distribution of DR grades revealed that 17 patients had mild NPDR, 5 had moderate NPDR, 3 had severe NPDR and none had proliferative DR. Table 1 shows the patients’ baseline characteristics: the data reveal a significant difference between the two groups (no DR and DR) regarding type of DM, age, years since diagnosis and years of insulin treatment. Among patients with DR, a higher percentage had T2D. The mean age was higher in the group without DR (52.3 versus 44.7 years), while the median time since diagnosis was longer in the group with DR (median: 14 versus 1 year). Additionally, more patients were on insulin treatment in the DR group (10% versus 7%), and the median duration of insulin treatment was also longer in the DR group (median: 15.5 versus 0 years).

**Table 1:** Baseline characteristics of the studied population associated with the presence or absence of DR.

	No DR *n* = 65	DR *n* = 25	*p* value
Type of diabetes			
T1D (%)	5 (7.7)	7 (28.0)	**0.018**
T2D (%)	60 (92.3)	18 (72.0)	
Gender			
Male, *n* (%)	42 (64.6)	19 (76.0)	0.301
Female, *n* (%)	23 (35.4)	6 (24.0)	
Age, [yrs, mean (SD)]	52.3 (13.0)	44.7 (14.7)	**0.016**
Years since diagnosis, [median (range)]	1.0 (0.0–20.0)	14.0 (0.0–34.0)	**< 0.001**
BMI≥25, *n* (%)	51 (78.5)	15 (60.0)	0.076
Diabetes medication			
Insulin, *n* (%)	7 (10.8)	10 (40.0)	**0.005**
Years of insulin, [median (range)]	0.0 (0.0–11.0)	15.5 (1.0–34.0)	**0.019**
OAM^*^ *n* (%)	45 (69.2)	16 (64.0)	0.634
OAM + Insulin, *n* (%)	2 (9.1)	2 (18.2)	0.586
GLP analogues^**^, *n* (%)	4 (7.3)	3 (14.3)	0.387
Cholesterol-lowering medications, *n* (%)	22 (33.8)	9 (36.0)	0.847
Antihypertensive medications, *n* (%)	33 (50.8)	8 (32.0)	0.109

^*^OAM: other antidiabetic medication. ^**^Glucagon like peptide-1 receptor analogues. *P*-values shown in bold indicate statistically significant differences between patients with no DR and those with DR.

Table 2 presents the distribution of the different grades of DR, DME and diabetic maculopathy at baseline, and after one, two and 3 years, along with the number of patients lost to follow-up. At baseline, 6 six patients (6.7%) had VTDR and were therefore not followed within the screening system anymore. All of the patients with severe NPDR also had DME, however, only three out of six patients with DME had severe NPDR. During the follow-up period, an additional three patients (3.3%) developed VTDR. In total, nine patients (10.0%) exited the screening due to VTDR before the final follow-up examination took place after 3 years. The prevalence of DR was 27.8% at baseline and fluctuated from 29.3 to 24.6% throughout the study. The presence of diabetic maculopathy was highest at baseline (20.0%) and varied from 13.7 to 14.7% during the follow-up examinations. At baseline, we also found the highest prevalence of DME to be 6.7%, and only two patients developed DME during follow-up.

**Table 2:** Distribution of the different grades of DR, DME and diabetic maculopathy at baseline, and after 1, 2 and 3 years, along with the number of patients lost to follow-up.

Diagnosis	Baseline *n* = 90 (%)	Screening (follow-up examination)		
		Year 1 *n* = 75 (%)	Year 2 *n* = 73 (%)	Year 3 *n* = 69 (%)
No DR	65 (72.2)	53 (70.7)	55 (75.3)	52 (75.4)
Mild NPDR^*^	17 (18.9)	18 (24.0)	13 (17.8)	12 (17.4)
Moderate NPDR	5 (5.6)	3 (4.0)	5 (6.8)	5 (7.3)
Severe NPDR	3 (3.3)	1 (1.3)	–	–
Diabetic macular edema^**^	6 (6.7)	–	2 (2.7)	–
Diabetic maculopathy^**^	18 (20.0)	11 (14.7)	10 (13.7)	10 (14.5)
Lost to follow-up		7 (7.8)	1 (1.3)	4 (5.4)

^*^NPDR: non-proliferative diabetes retinopathy; ^**^In at least one eye.

In terms of loss-to-follow-up, this comprised a total of 12 (13.3%) patients including one deceased patient. For the majority [seven patients (7.8%)], this loss to follow-up occurred after the baseline examination and at the time of the COVID-19 pandemic. Three patients underwent only 2 follow-up examinations, with their final examination occurring after 3 years of follow-up. A total of 69 patients (76.7%) completed the final examination after 3 years of follow-up.

Note: 3 patients had only two follow-up examinations, 1 patient after 1 and 3 years, and 2 patients after 2 and 3 years.

At baseline, 6 patients (6.7%) had VTDR, and 7 patients (7.8%) were lost to follow-up before the 1-year examination, leaving 77 (85.6%) patients who were followed beyond baseline. Of these patients, 19 patients (24.7%) either developed DR or showed a worsening in the severity of DR, and further 5 patients were lost to follow-up before completing the 3-year assessment. One patient became pregnant after 2 follow-up examinations, and was more frequently followed up and showed improvement in DR, from mild to no DR.

**Figure 1:**
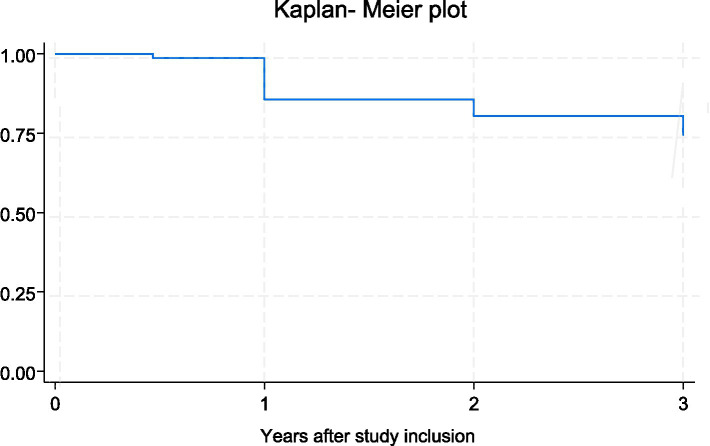
Kaplan–Meier plot showing the time to development or progression of DR in the study population.

Figure 1 presents the Kaplan–Meier plot for the time to development or progression of DR, with the development of DME also considered as DR progression.

The Cox regression analysis of the time to development or progression of DR revealed a significant association with the duration of DM and urine albumin/creatinine ratio (Table 3). For each additional year of diabetes, the risk of DR progression increased by 6.6% (HR = 1.066, *p* = 0.010), and for each unit increase in the urine albumin-to-creatinine ratio, the risk increased by 3.1% (HR = 1.031, *p* = 0.011).

**Table 3:** Univariable Cox regression analysis of factors associated with development or progression of diabetic retinopathy.

Variable	Hazard ratio	95% confidence interval	*p* value
Age per 1 year	1.010	0.98–1.04	0.530
Male gender	1.687	0.61–4.68	0.316
HbA1c per 11 mmol/mol	1.017	1.00–1.04	0.107
Total cholesterol per mmol/l	1.008	0.65–1.58	0.970
Urine creatinine per mmol/mol	0.955	0.89–1.03	1.222
Urine albumin per mg/l	1.000	0.995–1.004	0.939
Urine album-to-creatinine ratio per mg/mmol	1.031	1.01–1.06	**0.011**
Albuminuria (yes/no)	0.696	0.20–2.39	0.564
BMI per 1 kg/m^2^	0.950	0.88–1.02	0.162
Increase in duration of diabetes of 1 year	1.066	1.02–1.12	**0.010**
HT (yes/no)	0.825	0.30–2.30	0.712
SBP per 1 mmHg	1.000	0.97–1.03	0.975
DBP per 1 mmHg	1.007	0.97–1.05	0.725
MAP per mmHg	1.005	0.97–1.04	0.808
Type DM (1 vs. 2)	0.849	0.20–3.68	0.827
Smoking (yes/no)	1.607	0.58–4.46	0.362
Use of smokeless tobacco (yes/no)	0.574	0.08–4.30	0.588
Alcohol (> 21 units per month)	0.825	0.19–3.58	0.797
BCVA per logMar	0.482	0.003–69.282	0.774
IOP per mmHg	0.916	0.77–1.10	0.336
MOPP per mmHg	1.014	0.96–1.07	0.644

*P*-values shown in bold indicate a statistically significant association.

**Figure 2:**
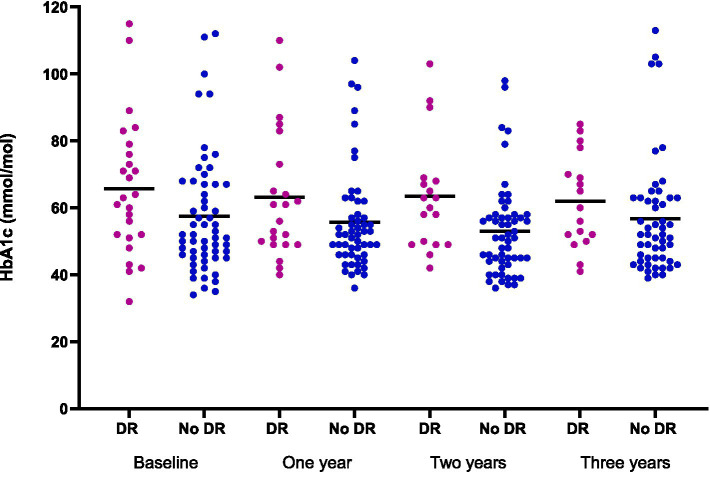
Group scatter plot of HbA1c values at baseline and annual follow-up for patients with and without DR. The horizontal lines represent the median within each group.

The Cox regression analysis found no significant association between the development or progression of DR and rising HbA1c levels. However, the grouped scatter plot (Figure 2) indicated a tendency of higher HbA1c values in the group with DR. When HbA1c was used as a time-varying covariate, a 1-unit increase was associated with a 2.5% higher hazard of DR progression (HR= 1.025, 95% CI 1.0035-1.0477).

## Discussion

To guide the development of the national DR screening program a pilot study was conducted to evaluate the prevalence and progression of DR and its associated risk factors among adults with diabetes in the Oslo region. Twelve patients with T1D and 78 patients with T2D were included. This distribution is representative for the general diabetes population in Norway, where approximately 90% are having T2D (13). At baseline, the prevalence of DR was 27.8% and fluctuated from 29.3 to 24.6% throughout the study. This is in line with the meta-analysis of 59 population-based studies that reported a global prevalence of DR at 22.3% among individuals with DM (2). In the US, the prevalence was estimated to be 26.43% (19), while another meta-analysis indicated a prevalence of 25.7% for the European region (20). The relatively stable prevalence of DR over the 3-year follow-up, despite a quarter of patients showing progression, is an important finding of this study. This outcome can be partially explained by the omission of patients with VTDR from further participation in the screening program after their condition was identified. The stable prevalence rates could also reflect the early detection and management of DR in this cohort, demonstrating the potential effectiveness of systematic screening programs in mitigating disease progression. As shown in the Kaplan–Meier plot, most cases of development or progression of DR in our cohort occurred within the first year of follow-up, highlighting the importance of early enrollment and continuous monitoring of patients with diabetes.

The significant association between DR progression and the duration of diabetes is well-documented in the literature and reaffirmed by our findings (21–23). Longer diabetes duration reflects cumulative exposure to hyperglycemia and other metabolic derangements, which drive microvascular damage. Additionally, the association between DR progression and the urine albumin-to-creatinine ratio highlights the systemic vascular implications of diabetes. Increased albuminuria is a marker of endothelial dysfunction (24, 25), and its link to DR progression underscores the interconnected nature of diabetic complications across organ systems (26, 27). These findings support the inclusion of urine albumin-to-creatinine ratio and diabetes duration as a routine parameter in DR risk stratification and screening protocols towards preventing DR progression.

Interestingly, while no statistically significant association between HbA1c levels and DR progression was observed, the time-varying analysis revealed an increase in the hazard for each 1-unit increase in HbA1c duren given time intervals. This finding is somewhat surprising, as glycemic control is widely regarded as fundamental in DR prevention. Previous studies, including the landmark Diabetes Control and Complications Trial (DCCT), have demonstrated a strong link between poor glycemic control and DR progression (28–32). However, the lack of significance in our study may be attributable to several factors, including the relatively short follow-up period and possible well-controlled/low fluctuating HbA1c over time. A relatively limited sample size, high proportion of newly diagnosed patients, along with the omission of patients with at least VTDR from further follow-up in the screening program, may also have influenced the results. Additionally, including patients with relatively good baseline glycemic control might have diluted the effect. Future studies should consider longitudinal tracking of HbA1c variability rather than relying solely on static baseline or follow-up values.

Another noteworthy finding is the lack of significant associations between DR progression and other commonly cited risk factors, such as gender, hypertension or BMI. While these factors are often implicated in the development and progression of DR (33–36), their role may vary depending on population characteristics and study design. For example, the relatively small sample size may have limited the statistical power to detect these associations. Furthermore, the uniformity of care provided within the study may have mitigated some of these risk factors, such as uncontrolled HT, which is modifiable through intervention.

The relatively low incidence of VTDR during follow-up (4%) is an encouraging finding. It suggests that most patients with mild or moderate NPDR at baseline may not progress to vision-threatening stages within 3 years. This has significant implications for screening protocols and supports consideration of extended screening intervals in low-risk individuals (37, 38), including those with no detectable DR or mild NPDR, relatively short duration of T2D, good metabolic control, well-controlled blood pressure, and no evidence of DME.

Implementing risk stratification based on parameters such as diabetes duration, albumin-to-creatinine ratio, and DR severity could optimize resource allocation, particularly in healthcare systems with limited capacity for frequent screenings (39). The challenges posed by patient attrition during the study period highlight an area for improvement in longitudinal DR screening. Approximately 13.3% of patients were lost to follow-up, with the majority dropping out during the COVID-19 pandemic at year 1. COVID-19 related loss to follow-up was primarily attributed to fear of attending hospital appointments due to the perceived risk of COVID-19 infection during transportation or while in the clinic. This underscores the need for robust patient engagement strategies, particularly during external disruptions. Telemedicine, remote monitoring, including community-based fundus imaging and OCT acquisition, as well as optometrist-driven follow-up and AI-assisted remote imagine grading, together with digital communication platforms could play a critical role in improving follow-up rates and ensuring continuity of care in similar settings (10, 40–42). Additionally, targeted education and outreach programs could address barriers to participation, such as lack of awareness or logistical challenges (40, 42–44). Nearly one-quarter of patients developed DR or experienced disease worsening. Patients with VTDR were not followed further within the screening system and may have underestimated the true progression rates. Moreover, around 90% of the participants followed-up from baseline had either no DR or mild NPDR. This underscores that variations in study populations, definitions of progression and endpoints, as well as differences in screening intervals and follow-up time, make study comparisons challenging.

The study also highlights the importance of addressing health disparities in DR screening and management. While Norway offers a well-developed healthcare system, the lack of a fully implemented systematic DR screening program can lead to gaps in care, particularly for patients not regularly followed by ophthalmologists. The establishment of regional screening programs nationwide could improve early detection rates and ensure consistent follow-up, ultimately reducing the burden of vision-threatening DR (5–7).

Finally, although these findings provide valuable insights, they should be interpreted in the context of the study’s limitations. As a pilot study, the relatively small sample size and single-center design limit both generalizability and statistical power. In addition, the low baseline prevalence of DR is likely influenced by selection bias inherent to a referral-based screening program. Underserved or higher-risk populations may therefore be underrepresented, limiting the applicability of prevalence estimates to the broader diabetic population in Norway. Future multicenter studies with larger sample sizes and longer follow-up are needed to validate these findings and to refine DR screening protocols.

Overall, this study contributes to the growing body of evidence supporting systematic DR screening as an effective tool for early detection and management (5–7). By identifying key risk factors for DR progression and demonstrating the potential for extended screening intervals, our findings provide a foundation for developing evidence-based guidelines tailored to the Norwegian healthcare context. Future research should focus on optimizing screening intervals, integrating risk stratification, and exploring innovative approaches to enhance patient engagement and retention.

In conclusion, findings from this pilot DR screening program support the development of a national, systematic screening approach as an effective clinical tool for the early detection and management of vision-threatening complications. By identifying risk factors for progression and demonstrating that low-risk patients may safely undergo extended screening intervals, this program provides a framework for evidence-based DR screening in Norway. Future efforts should focus on optimizing screening intervals, applying risk-based stratification, and enhancing patient engagement to maximize clinical impact.

## Data Availability

The raw data supporting the conclusions of this article will be made available by the authors, without undue reservation.
